# Total elbow arthroplasty with tricep turn-down flap in an old unreduced elbow dislocation: A case report

**DOI:** 10.1016/j.ijscr.2023.108432

**Published:** 2023-06-21

**Authors:** Renaldi Prasetia, Siti Zainab Bani Purwana, Nucki Nursjamsi Hidajat, Hermawan Nagar Rasyid

**Affiliations:** a Department of Orthopaedics – Traumatology, Universitas Padjadjaran, Hasan-Sadikin General Hospital, Bandung, Indonesia; b Faculty of Medicine, Universitas Padjadjaran, Hasan-Sadikin General Hospital, Bandung, Indonesia

**Keywords:** Neglected elbow dislocation, Total elbow arthroplasty, Turn-down flap, Tricep turn-down flap, Case report

## Abstract

**Introduction and importance:**

The treatment preference for neglected elbow dislocation is by open reduction and lengthening the tricep muscle. If the dislocation is not reduced for more than 6 months, degenerative resorption would have occurred. We did total elbow arthroplasty (TEA) with a tricep turn-down flap for the management in this case. The use of a tricep flap for tricep shortening after unreduced elbow dislocation has been reported in previous publications but none used a turn-down flap.

**Case presentation:**

An 82-year-old woman came to the orthopedic outpatient clinic with pain and discomfort on her right elbow. The arm affected by the injury was her dominant hand, restricting her from farming and leisure activities. Exploration findings confirmed the presence of a dislocated elbow with associated soft tissue complications. The cartilage was degeneratively destructed, and the tricep muscle was retracted.

**Clinical discussion:**

In our case, tricep shortening was managed with elongation using a turn-down flap. The lack of soft tissue layers and thin fibrous fascias results in tension-vulnerable TEA surgical wounds. This is caused by the insufficient coverage of the joint, which leads to wound complications. Previous studies of turn-down flap procedures showed good flap survival and functional outcomes.

**Conclusion:**

Tricep turn-down flap could be an option for tricep lengthening procedures in unreduced neglected elbow joint dislocation.

## Highlights

• One of the difficulties in treating neglected elbow dislocation is contracture or soft tissue shortening.
• TEA with a tricep turn-down flap for the treatment of neglected elbow joint dislocation results in improved outcomes.
• The lack of soft tissue layers and thin fibrous fascias results in tension-vulnerable TEA surgical wounds.

## 1 Introduction

Elbow dislocation is the second most prevalent dislocation in adults [1]. A neglected case of elbow dislocation is often found in underdeveloped and developing countries because of low educational level, low socioeconomic status, lack of awareness, and unavailability to access properly qualified specialists [1,2]. Difficulties in treating neglected elbow dislocation are contracture or soft tissue shortening, ligament fibrosis, and insufficiency, nerve injury, and myositis ossificans. Patients' incompliance could also be a hindrance [1]. If injuries causing elbow dislocations are not managed with adequate treatment, the elbow could be chronically unstable, causing recurrent dislocation [3]. The stability of the elbow is maintained by dynamic and static stabilizers. Muscles crossing the elbow contribute to elbow stability as dynamic stabilizers [4].

After 3 months of unreduced dislocation, fibrosis, cartilage degeneration, and regional osteoporosis become extensive [1]. If the dislocation is not reduced for more than 6 months, degenerative resorption would have occurred, where total, excision, or replacement arthroplasty is needed [1,2].In addition, 1.5 % of total elbow arthroplasty (TEA) had elbow dislocation as the indication [5]. The use of a tricep flap for tricep shortening after unreduced elbow dislocation has been reported in previous publications but none used a turn-down flap [1,6].

This study presents a case of neglected unreduced elbow dislocation treated with TEA and a tricep turn-down flap. The neglected dislocation was complicated by degenerative destruction of the cartilage and tricep muscle shortening. We aim to review the short-term outcome of the procedure seen clinically and subjectively. This work has been reported in line with the SCARE guidelines [7].

## 2 Case presentation

An 82-year-old woman came to the orthopedic outpatient clinic with pain and discomfort on her right elbow. The arm affected by the injury was her dominant hand, restricting her from farming and leisure activities. Seven months before admission, the patient suffered from an injury to the elbow caused by falling with an outstretched arm. The patient then went to a primary doctor and was referred to the nearest hospital. On the third day of admission, she was referred again to a third-referral hospital in the city because of lack of facilities. She was diagnosed with elbow dislocation and suggested to undergo open reduction by the orthopedic surgeon but the patient declined. The patient sought treatment from a bonesetter, and massages were performed to the injured elbow. Complaints did not subdue, and the patient felt numbness to the affected elbow. The patient eventually sought medication again at a hospital, but owing to complications from neglect and bonesetter treatment, she was referred to an orthopedic specialist with expertise in shoulders and elbows in a third-referral hospital. We performed open reduction to the dislocated elbow but failed on the third week postoperation. The patient was then scheduled for a TEA. The procedure was further delayed by a waiting period for the national health insurance in our hospital to approve the procedure. Seven months after the injury, the procedure had been approved to be performed. At the time, the patient's range of motion (ROM) in elbow flexion and elbow extension was 62.3° with 0° movement arc. Supination and pronation was not measured because the patient was unable to reach neutral position.

**Fig. 1:**
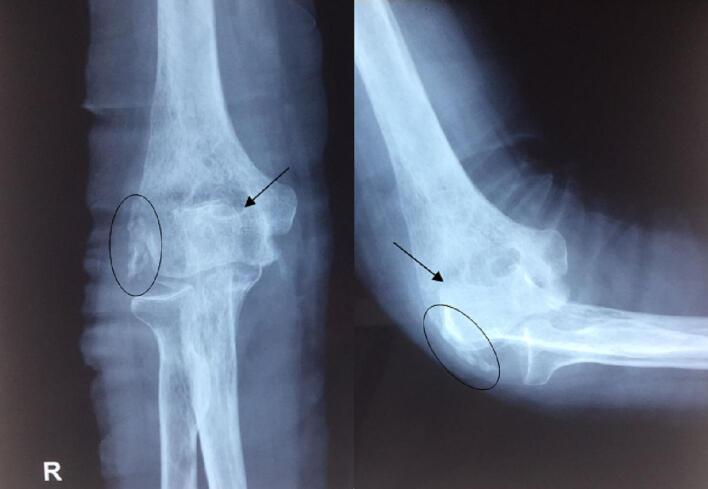
Right anteroposterior and lateral elbow X-ray. Image showing neglected dislocation of the elbow joint (arrow) with soft tissue calcification (circle).

We conducted a physical examination. Right elbow varus deformity was found. The elbow was unstable due to traction by the retracted tricep. X-ray taken on the right elbow showed neglected right elbow joint dislocation and calcification of the soft tissues surrounding the elbow joint, indicating the presence of myositis ossificans. The right elbow joint was porotic as seen in the X-ray image (Fig. 1). The preoperative diagnosis was neglected right elbow joint dislocation.

## 3 Surgical technique

**Fig. 2:**
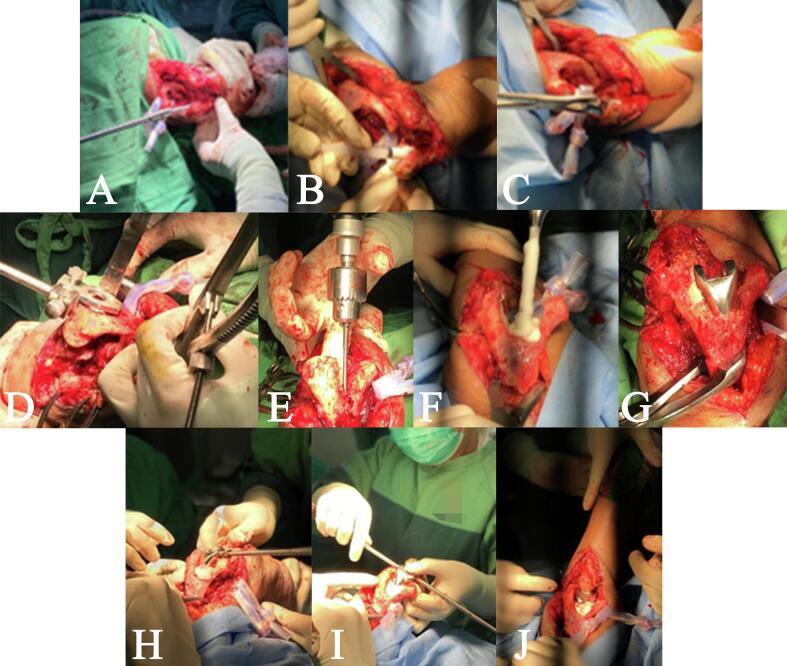
TEA operative procedure. (A) Ulnar broaching. (B) Cement insertion. (C) Insertion of the ulnar component. (D) Cutting block placement for osteotomy guiding. (E) Opening the medullary canal. (F) Cement insertion. (G) Insertion of the humeral component. (H-J) Reduction and implant assembly.

**Fig. 3:**
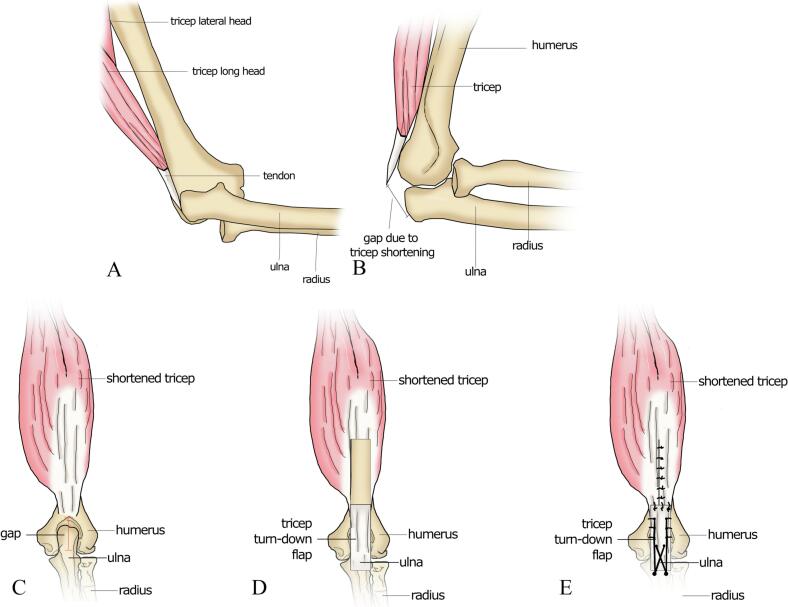
Illustration of the tricep turn-down flap procedure. (A) Medial view of an elbow dislocation in our patient. (B) Lateral view of reduced elbow dislocation with tricep shortening. (C) Posterior view of reduced elbow dislocation. (D) Turn-down of a tricep flap. (E) Flap anchoring and closure of the flap donor site.

**Fig. 4:**
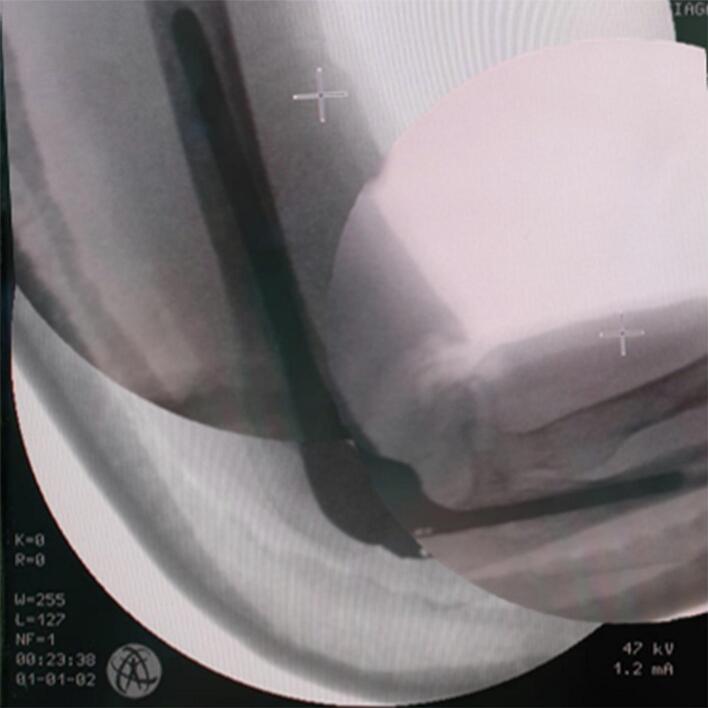
Intraoperative c-arm image.

**Fig. 5:**
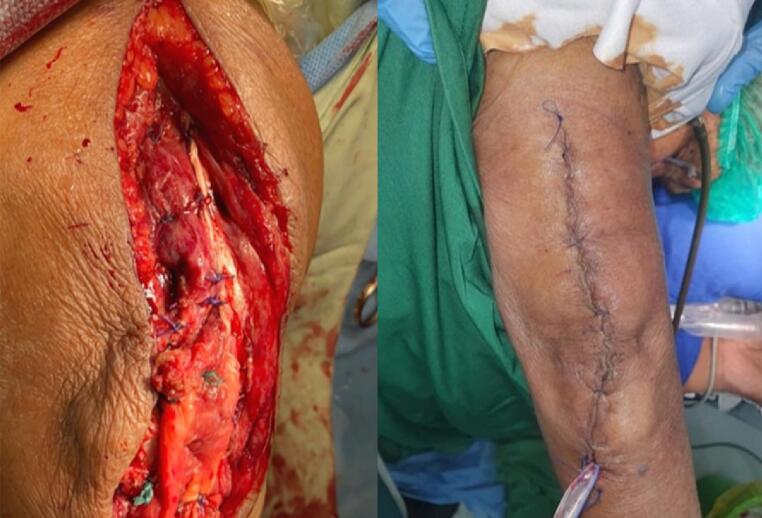
Wound closure.

With the long duration of unreduced dislocation and soft tissue involvement in considerations, we planned TEA with a tricep turn-down flap for the treatment in this patient. The procedure was performed with the patient put under general anesthesia. The patient was placed in the lateral decubitus position, with the contralateral arm 90° elevated and the contralateral elbow 90° flexed. Exploration findings confirmed the presence of a dislocated elbow with associated soft tissue complications. The cartilage was degeneratively destructed, and the tricep muscle was retracted, pulling its ulnar insertion, and constricting flexion. TEA was performed with ulnar nerve preservation (Fig. 2). The tricep was cleared out of the joint to provide visualization of the articular surfaces. As shown in the illustration (Fig. 3), the turn-down flap of the tricep was made to elongate the shortened tricep with longitudinal incisions, making a flap long enough to cover the elbow joint and reach the proximal ulna without restricting elbow flexion. The flap was then anchored. Once reduced, intraoperative movements of the elbow joint were assessed and intraoperative c-arm image was taken (Fig. 4). Wound closure was then performed using stitches (Fig. 5). The affected elbow joint was immobilized with a posterior splint in 90° flexion position. There were no significant difficulties or intraoperative complications.

## 4 Outcome and follow-up

**Fig. 6:**
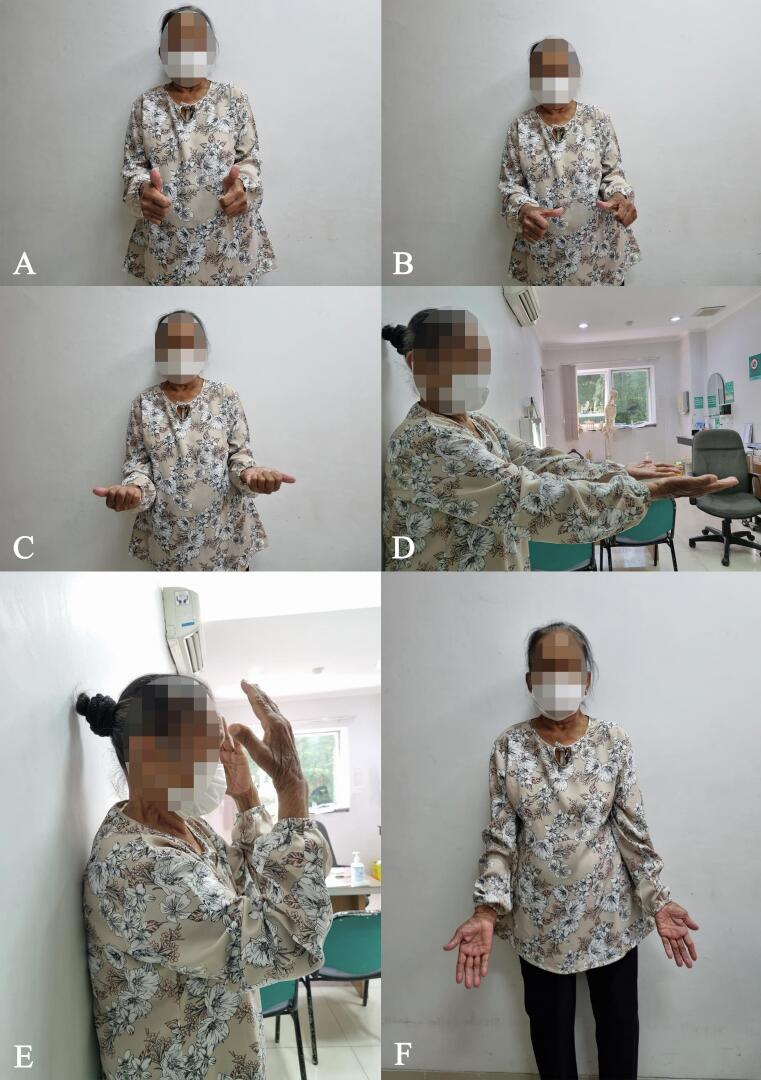
Patient's ROM 2 months postoperation. (A) Neutral rotation. (B) Pronation. (C) Supination. (D) Neutral extended elbow. (E) Elbow flexion. (F) Elbow extension.

Wound closure was achieved in 2 weeks. The splint was removed at 3 weeks after the surgery, and the patient was motivated to perform passive elbow joint motion. Active movement and object lifting of the elbow were allowed after 2 months. The patient started to perform normal activities 2 months after surgery. Functional outcomes taken 3 months after surgery showed improvements. The elbow joint was stable with normal movements and good ROM with 119.8° elbow flexion and 33° elbow extension, reaching an 86.8° movement arc (Fig. 6). Supination reached 79.5° ROM, and pronation reached 31.2° ROM. Visual analog scale (VAS) scores 3 months after the procedure were better, from 6 preoperatively to 2 postoperatively. The American Shoulder and Elbow Surgeons (ASES) score and disabilities of the arm, shoulder, and hand (DASH) score improved from 23 to 75 and from 66.7 to 26.7, respectively (Table 1). The patient was satisfied with the functional outcome. The patient had no signs of wound infection or other complications observed in 3 months postoperation.

**Table 1:** Patient's pain and functional assessment.

No	Assessment tool	Preoperative	3 months follow-up
1	VAS score	6	2
2	ASES score	23	75
3	DASH score	66.7	26.7
4	ROM		
	a. Elbow flexion	62.3°	119.8°
	b. Elbow extension	62.3°	33°
	c. Supination	n/a	79.5°
	d. Pronation	n/a	31°

## 5 Clinical discussion

TEA was originally developed for advanced rheumatoid arthritis [8,9]. Currently, trauma sequels, and recurrent elbow dislocations especially in elderly are included as indications of TEA [[8], [9], [10]]. The presence of soft tissue impairments in this case also supports the need of doing TEA [9].

Patients who lack tricep function and are unable to limit activities are relative contraindications for TEA. In our case, tricep shortening was managed with elongation using a turn-down flap [8]. The lack of soft tissue layers and thin fibrous fascias results in tension-vulnerable TEA surgical wounds. This is caused by the insufficient coverage of the joint, which leads to wound complications [9]. Tricep muscle restriction is indicated if intraoperative flexion is less than 80° [11].

Turn-down flaps are usually used as a part of the modified Lindholm technique to augment or repair Achilles tendon ruptures [12]. A case report by Lin et al. showed a quadricep tendon turn-down flap to augment a patellar tendon repair, with ROM exercises allowed in 3 weeks, immediately after cast removal [13]. A study by Gedam et al. reports cases where they performed a gastrocnemius turndown flap to fill the gap caused by Achilles tendon rupture, with ROM exercise, and weight-bearing started in 3 and 4 weeks, respectively [14]. Previous studies of turn-down flap procedures showed good flap survival and functional outcomes [[12], [13], [14], [15]].

Short-term outcomes of TEA in modern days are found to be satisfactory by restoring function, relieving pain, and improving elbow motion, with a 92 % 5-year survival rate and 20 %–40 % complication rate. Instability is found in 2 % of patients at 3 to 12 years after follow-up. This case report only presents the short-term outcomes of the patient, but the elbow examined on follow-ups showed good stability [8].

## 6 Conclusion

Neglected elbow joint dislocation for more than 6 months can cause soft tissue involvement. In this case, we did TEA as the management using a tricep turn-down flap to lengthen the contracted tricep muscle. In 2 months, our patient had good postoperative functional outcomes. A tricep turn-down flap can be an option for tricep lengthening procedures in unreduced neglected elbow joint dislocation.

## Disclaimer

Patient details are not included in the figures.

## Consent for publication

Informed consent for the publication of the data and images was provided in writing by the patient and guardian.

## Ethical approval

This study is a case report from an individual patient in which patient consent had been taken, with no patient detail included in the manuscript or figures. Therefore, this study is exempt from ethical approval in the Hasan Sadikin General Hospital.

## Funding

There was no funding provided by sponsors in the public, commercial, or nonprofit sectors.

## Author contribution

All authors contributed significantly to the work reported. Renaldi Prasetia contributed to the conception and study design; performed the operation, data collection, and analysis; drafted the manuscript; performed critical review; revised and created illustrations; and made the final approval for publishing. Siti Zainab Bani Purwana contributed to data collection and analysis, drafted the manuscript, created illustrations, and made the final approval for publishing. Nucki Nursjamsi Hidajat contributed to data collection and analysis, and final approval for publishing. Hermawan Nagar Rasyid contributed to performing the operation, data collection and analysis, and final approval for publishing.

## Guarantor

All authors; Renaldi Prasetia, Siti Zainab Bani Purwana, Nucki Nursjamsi Hidajat, and Hermawan Nagar Rasyid are the Guarantor of this study.

## Research registration number

The technique performed on the patient is a combination of pre-existing techniques, so we did not register this research.

## Declaration of competing interest

There is no conflict of interest in this work.
